# Understanding alternative splicing of Ca_v_1.2 calcium channels for a new approach towards individualized medicine

**DOI:** 10.1016/S1674-8301(10)60027-9

**Published:** 2010-05

**Authors:** Ping Liao, Tuck Wah Soong

**Affiliations:** a National Neuroscience Institute, 11 Jalan Tan Tock Seng, Singapore 308433; b Department of Physiology, Yong Loo Lin School of Medicine, National University of Singapore, Singapore 117597

## Abstract

Calcium channel blockers (CCBs) are widely used to treat cardiovascular diseases such as hypertension, angina pectoris, hypertrophic cardiomyopathy, and supraventricular tachycardia. CCBs selectively inhibit the inward flow of calcium ions through voltage-gated calcium channels, particularly Ca_v_1.2, that are expressed in the cardiovascular system. Changes to the molecular structure of Ca_v_1.2 channels could affect sensitivity of the channels to blockade by CCBs. Recently, extensive alternative splicing was found in Ca_v_1.2 channels that generated wide phenotypic variations. Cardiac and smooth muscles express slightly different, but functionally important Ca_v_1.2 splice variants. Alternative splicing could also modulate the gating properties of the channels and giving rise to different responses to inhibition by CCBs. Importantly, alternative splicing of Ca_v_1.2 channels may play an important role to influence the outcome of many cardiovascular disorders. Therefore, the understanding of how alternative splicing impacts Ca_v_1.2 channels pharmacology in various diseases and different organs may provide the possibility for individualized therapy with minimal side effects.

## INTRODUCTION

Calcium ions play a critical role in muscle function. Voltage-gated calcium channels (VGCCs) govern the depolarization induced Ca^2+^ entry in many excitable cells, such as neurons, cardiac and smooth muscle cells[1]. Of the 10 known VGCCs, L-type Ca_v_1.2 channel is the most widely expressed channel in the cardiovascular system and is essential for the contraction of heart and arterial smooth muscles. The T type Ca_v_3.1 and L type Ca_v_1.3 channels are expressed in the sinus node cells and modulate pacemaker activity[2].

VGCCs are composed of multiple subunits. The pore forming α_1_ subunit is the basic structure of the channel, while the β, α_2_δ and/or γ subunits interact with the α_1_ subunit and play a modulatory role. Calcium channel blockers (CCBs) are widely used in clinical practice to treat cardiovascular disorders from hypertension to angina pectoris, arrhythmia, Raynaud syndrome, and cerebral vasospasm, etc. The basic effect of CCBs is to inhibit VGCCs by binding to the pore forming α_1_ subunit and the Ca_v_1.2 channel is the major target of CCBs.

Three classes of small molecule CCBs are currently in clinical use: 1,4-dihydropyridines (DHPs), phenylalkylamines (PAAs), and benzothiazepines (BTZs). They all bind to the α_1_ subunit of Ca_v_1.2 channel[3],[4]. After several decades of development, new generations of CCBs are more selective on target organs with fewer side effects. For example, the second- and third-generation of DHPs exhibit higher vascular selectivity with less negative inotropic effect and sympathetic activation compared with the first-generation blockers. However, variable responses still exist among patients. One example is that elderly or black patients are more sensitive to CCBs than young and white patients[5],[6]. Such effects could be due to the presence of variable drug metabolizing enzymes, drug transportation systems or drug targets.

Genetic factors determine drug response taking into consideration many other factors such as age, sex, body weight, and heath status. Pharmacogenomics provides information on the linkage of genetic factors to drug responses and may also provide the basis for the use of safer and more efficient medications to patients. In hypertension, genetic associations with antihypertensive response have been established for diuretics, beta-blockers, ACE inhibitors and angiotensin1 receptor blockers. However, most of the information is lacking in calcium channel blockers. Recently, three single nucleotide polymorphisms (SNPs) of Ca_v_1.2 channel were identified to link with antihypertensive outcome[7]. Although pharmacogenomics is a useful tool to help understanding the variable response of drug sensitivity among patients with different genetic background, it cannot address the issue about the changes of drug response during the progress of a disease or development of a new disease. The response to drug of a patient could be different when he/she is healthy or sick. The patient can also respond by changing from a drug sensitive state to an insensitive state.

Alternative splicing is a post-transcriptional modification process. Multiple functional variants could be generated from a single gene. Recently, a large number of alternatively spliced exons have been identified within the pore-forming α_1_ subunit of Ca_v_1.2 channel[8]–[10]. In this review, we will discuss the dynamic regulation of alternative splicing of Ca_v_1.2 channels under physiological and pathophysiological conditions and the influence of such changes on pharmacology. The proteomic structure of Ca_v_1.2 channels could change under pathological conditions due to alternative splicing. The way we view individualized medicine in treating cardiovascular diseases may need to be expanded beyond pharmacogenomics.

## ALTERNATIVE SPLICING AND CCB BINDING

The human Cav1.2 gene, *CACNA1C*, codes for the α_1_ subunit and contains 55 exons. At least 19 exons are subjected to alternative splicing[8]–[10]. The distribution of the splice sites could be found in our previous review[9]. The number is increasing with reports of the discovery of new splice variants. Exon 34 was recently added to the list[11] and a novel exon 1C was reported to exist in rat arterial smooth muscles[12]. If there were a human exon 1C, total of 21 exons could undergo alternative splicing. Theoretically there will be 2[21] combinations. However, these splice variants are not expressed at the same level. Some alternatively spliced exons were found to be predominantly expressed in certain tissues[9],[13]–[15].

The binding site for CCBs is mainly composed of the transmembrane segments 5 and 6 (S5 and S6) of domains I to IV. By using photoaffinity labeling, antibody mapping, and chimeric study, DHPs were found to bind IIIS5, IIIS6 and IVS6 segments, while IIIS6 and IVS6 are the binding sites for PAAs and DTZs[16],[17]. IIIS5 segment was also suggested to participate in PAA inhibition[18] and IS6 in DHP inhibition[15]. Of these regions, IS6 is encoded by alternatively spliced exons 8 and 8a[15], while the rest of the binding sites are encoded by constitutive exons[9]. Although other alternatively spliced exons are not involved in drug binding, they can affect the channel sensitivity to CCBs by altering gating properties[13],[19].

## TISSUE SPECIFIC SPLICE VARIANTS CORRELATE WITH CCBS SENSITIVITY

The pharmacological effect of CCBs depends on their inhibition of Ca^2+^ influx through Ca^2+^ channels in cardiac and vascular smooth muscles. However, there exist variable responses to blockade of Ca_v_1.2 channels by CCBs within the two tissues. For example, vascular smooth muscles are more sensitive to DHPs than cardiac muscles. One obvious reason is that calcium channels in smooth muscle possess a higher binding affinity than in cardiac muscle[20]. The second reason is that vascular smooth muscles have a more depolarized membrane potential than cardiac cells[21],[22] and as such more Ca_v_1.2 channels are locked in an inactivated state which favors the DHP block[19]. Recently, the difference in the molecular structures within cardiac and smooth muscles generated by alternative splicing has emerged as a third determinant factor for CCBs block[13].

Ca_v_1.2 channel is generally divided into a cardiac isoform (Ca_v_1.2a) and a smooth muscle isoform (Ca_v_1.2b). Cav1.2a channel is the predominant channel in heart while Ca_v_1.2b channel in smooth muscles. Ca_v_1.2a channel contains the combination of exons 1a/8a/-9*/32/33[13],[23], while the smooth muscle form (Ca_v_1.2b) contained exons 1b/8/9*/32/33[24]. Exon 1b was named exon 1 in previous reports. Recently, an exon 1c was cloned from rat cerebral arteries and it was reported to be the predominant exon in smooth muscles[12]. However, the human exon 1c has not yet been discovered.

The Ca_v_1.2b channel is more sensitive to DHP block than Ca_v_1.2a channel which is similar to the observations in native heart and blood vessels[15],[25],[26]. The molecular component for drug sensitivity was shown to be determined by the inclusion or exclusion of the mutually exclusive 8 and 8a exons that encode the IS6 transmembrane segment. Cav1.2 channels containing exon 8 is more sensitive to isradipine than channels containing exon 8a[15]. An early report showed that IS6 region is important for channel inactivation properties[27]. However, both Cav1.2a and Cav1.2b channels share similar activation and inactivation properties[15],[25]. Thus, exons 8 and 8a were believed to affect DHP sensitivity through altering binding affinity rather than changing the inactivation properties of the channels[15]. Besides Cav1.2b channel, there exists a small population of channels in blood vessels named Cav1.2SM channel with exon 33 deletion. The altered inactivation property of Cav1.2SM channel directly affects the channel's sensitivity to DHP[13].

Ca_v_1.2 channel activity is also regulated by phosphorylation[1],[28]–[30]. The N-terminal region of Cav1.2 channel is the target for protein kinase C[1],[31],[32]. Exon 1a from cardiac isoform Cav1.2a channel contains two threonine sites at 27 and 31, and they are not present in smooth muscle Cav1.2b channel. There also exists a potential protein kinase A site within the alternatively spliced exon 9* within I-II loop[33]. However, it is unknown whether phosphorylation of the putative serine/threonine kinase sites found in the alternatively spliced exons might affect the sensitivity of cardiac or smooth muscle Cav1.2 channels to CCBs.

Although there exist predominant Ca_v_1.2 channels in heart and blood vessels, numerous splice variants are found to be expressed in cardiovascular system[34]. The presence of splice variants with lower expression could be of particular importance in physiology and pharmacology. For example, the deletion of exon 33 in a small population of Ca_v_1.2 channels in arterial smooth muscles relates with the left shifted window currents recorded in native smooth muscles[13],[35]. The DHP sensitivity was altered due to the changes of gating properties[13]. Other alternative spliced exons could also exhibit various CCB sensitivities. Mutually exclusive exon 31 at IVS3 region is more sensitive to DHPs block than exon 32[36]. Mutually exclusive exon 21 encoding IIIS2 segment is less sensitive to DHP block than 22[36],[37]. The results from 65 human heart samples showed the presence of a large number of alternative spliced exons within individual heart tissues[38]. Two human hearts expressed unusually high level of exon 8 instead of exon 8a. This information is of particular importance as exon 8 determines the higher sensitivity of blood vessels to DHP block. Abnormal expression of exon 8 in heart will generate critical side effect in heart if DHPs are used to treat hypertension in these patients. This data therefore underlies the importance of understanding the splicing profiles in individual patients.

## ALTERNATIVE SPLICING AND CARDIOVASCULAR DISORDERS

Ca_v_1.2 channels are crucial for cardiovascular functions as deletion of the gene in mouse leads to embryonic lethality[39]. Alternative splicing of Ca_v_1.2 channels was linked to many diseases[40]. Mutations of Ca_v_1.2 gene was reported in Timothy syndrome, a disorder characterized by dysfunction in multiple organ systems, including heart, skin, eyes, teeth, immune system and brain[41],[42]. Patients usually die at an early age from lethal arrhythmia. The mutations are found at the mutually exclusive exons 8 and 8a and two mutations were found: G406R and G402S. Patients with G406R at exon 8 have a milder symptoms compared with patients with G406R and/or G402S at exon 8a. It should be noted that the exons 8/8a mentioned in the above two papers refer to exons 8a/8 respectively in other reports[9]. Channel inactivation properties are impaired by the mutations. As a consequence, a continuing influx of Ca^2+^ ions will result in the lengthening of action potential, leading to cardiac arrhythmia and sudden death. The levels of expression of exon 8 and 8a is different in various organs and tissues and thus the location of the mutations in exon 8 or 8a would determine the severity of the symptoms and the involvement of other organs. CCBs are ideal to treat the patients by reducing the Ca^2+^ influx from mutant channels.

Alternative splicing of Ca_v_1.2 channels has identified to be altered in cardiovascular disorders. Mutually exclusive exons 31 and 32 are developmentally regulated[43] and reemergence of fetal exon was found in hypertrophied or failing hearts[44],[45]. Gidh-Jain *et al*[44] reported the switch to a fetal exon in the hypertrophied rat hearts 21 days post myocardial infarction. Yang *et al*[45] reported the increased expression of fetal exon in human failing hearts. Furthermore, a number of exons were found to be altered in vascular smooth muscles of patients with atherosclerosis[11]. Exon 9* was absent in blood vessels from patients while exon 21 was expressed in healthy arteries, but in patients a switch in expression to the mutually exclusive exon 22 was observed in almost all atherosclerotic arteries examined. Exon 41a was also expressed exclusively in normal arteries. In another report, alternative splicing profiles underwent changes in rats with hypertension. Such changes occurred at multiple splicing sites generating many splice variants[46]. We recently reported the alternative splicing of a number of exons was remodeled in a rat model of myocardial infarction[47]. The remodeling mainly occurred in the infarct area. In contrast to the predominant channels expressed in normal heart, channels with novel combinations of exons appeared in heart with myocardial infarction. Importantly, the alteration of channels in myocardial infarction, hypertension, and atherosclerosis exhibited channel properties changes by electrophysiology studies[11],[46],[47]. Such changes would potentially have great impact on CCBs sensitivity.

## PERSPECTIVES AND CHALLENGES

The progress in the study of alternative splicing of Ca_v_1.2 channels highlights a novel way towards individualized medication. Besides SNPs, post transcriptional modification produces Ca_v_1.2 channels with huge variability both in structure and function. Each person could express slightly different splice variants in different tissues. But the functional impact could be enormous. Furthermore, under pathological conditions, the splice patterns can be altered. Such alteration could be variable at different stages of the disease. Thus, each patient could express a signature pattern of Ca_v_1.2 channels generated by alternative splicing. This provides possible targets for individualized medication. However, many questions need to be addressed first and chief of which is how the splicing profile from different organs of a patients can be achieved. The nature of alternative splicing makes it impossible to get such information simply from blood. Also the length of the gene and multiple splicing sites makes it difficult to determine combinatorial profiles for the expression of the many alternatively spliced exons in the full length Ca_v_1.2 channel transcripts. The next obstacle is to select suitable splice variants as targets for drug discovery and development. Most of the current CCBs in use are not designed against one splice variant without affecting others. The understanding of alternative splicing of Ca_v_1.2 channels is far from complete. One example is the hemichannels generated by misspliced exons[48]. Hemichannels in other channels were found to relate with congenital disease[49]. The role of hemichannels or aberrant channels in Ca_v_1.2 channels remains mostly unclear.

In this review, we discussed the progress in relating alternative splicing of Ca_v_1.2 channels to cardiovascular pharmacology and pathophysiology. However, the knowledge in other organs and systems are mostly lacking. For example, the splicing pattern in nervous system is not well studied. Considering the higher expression of Ca_v_1.2 channels in neurons, CCBs in treating nervous system disorders could attract more attention if neuronal specific CCB is discovered one day in the future. In conclusion, we presented another consideration for the development or discovery of drugs against Ca_v_1.2 channels that may be efficacious in the management of cardiovascular disorder.
